# Aquaporin-4 expression in distal myopathy with rimmed vacuoles

**DOI:** 10.1186/1471-2377-12-22

**Published:** 2012-04-27

**Authors:** Akihiko Hoshi, Teiji Yamamoto, Saeko Kikuchi, Tomoko Soeda, Keiko Shimizu, Yoshikazu Ugawa

**Affiliations:** 1 Department of Neurology, Fukushima Medical University, 1, Hikarigaoka, Fukushima, 960-1295, Japan; 2 JST, Research Seeds Program, Fukushima, Japan

**Keywords:** Distal myopathy with rimmed vacuoles (DMRV)/hereditary inclusion body myopathy (hIBM), Rimmed vacuoles (RVs), Aquaporin-4 (AQP4), Fast-twitch type 2 fiber

## Abstract

**Background:**

Distal myopathy with rimmed vacuoles/hereditary inclusion body myopathy is clinically characterized by the early involvement of distal leg muscles. The striking pathological features of the myopathy are muscle fibers with rimmed vacuoles. To date, the role of aquaporin-4 water channel in distal myopathy with rimmed vacuoles/hereditary inclusion body myopathy has not been studied.

**Case presentation:**

Here, we studied the expression of aquaporin-4 in muscle fibers of a patient with distal myopathy with rimmed vacuoles/hereditary inclusion body myopathy. Immunohistochemical and immunofluorescence analyses showed that sarcolemmal aquaporin-4 immunoreactivity was reduced in many muscle fibers of the patient. However, the intensity of aquaporin-4 staining was markedly increased at rimmed vacuoles or its surrounding areas and in some muscle fibers. The fast-twitch type 2 fibers were predominantly involved with the strong aquaporin-4-positive rimmed vacuoles and TAR-DNA-binding protein-43 aggregations. Rimmed vacuoles with strong aquaporin-4 expression seen in the distal myopathy with rimmed vacuoles/hereditary inclusion body myopathy patient were not found in control muscles without evidence of neuromuscular disorders and the other disease-controls.

**Conclusions:**

Aquaporin-4 might be crucial in determining the survival or degeneration of fast-twitch type 2 fibers in distal myopathy with rimmed vacuoles/hereditary inclusion body myopathy.

## Background

Autosomal recessive distal myopathy with rimmed vacuoles (DMRV)/hereditary inclusion body myopathy (hIBM) is clinically characterized by preferential involvement of the distal leg muscles in the very early stage, and later of most proximal muscles. One of the striking pathological features of this myopathy is muscle fibers with rimmed vacuoles (RVs) [1,2]. DMRV/hIBM is caused by a mutation in the uridine diphosphate-*N*-acetylglucosamine 2-epimerase/*N*-acetylmannosamine kinase (*GNE*) gene, which encodes a bifunctional enzyme catalyzing the 2 exclusive rate-limiting reactions of sialic acid synthesis in the cytosol [3,4]. However, why these mutations produce a myopathy with RVs remains to be determined.

Aquaporin-4 (AQP4) is the main water channel of the neuromuscular system. In the skeletal muscle, AQP4 is predominantly localized to the sarcolemma of fast-twitch type 2 fibers [5-7]. AQP4 expression in the muscles is markedly reduced in patients with dystrophinopathy, dysferlinopathy, and amyotrophic lateral sclerosis (ALS) [8-10], but the pathophysiology underlying the reduction in expression is unclear. Changes in AQP4 expression, however, have not been studied in DMRV/hIBM thus far. In this communication, we aimed to characterize AQP4 expressions in the muscle fibers of a patient with DMRV/hIBM associated with *GNE* mutation. Furthermore, we investigated accumulation of TAR-DNA-binding protein-43 (TDP-43), a pathological hallmark of vacuolar myopathies [11,12], in the muscle fibers of DMRV/hIBM patients.

## Case presentation

**Figure 1:**
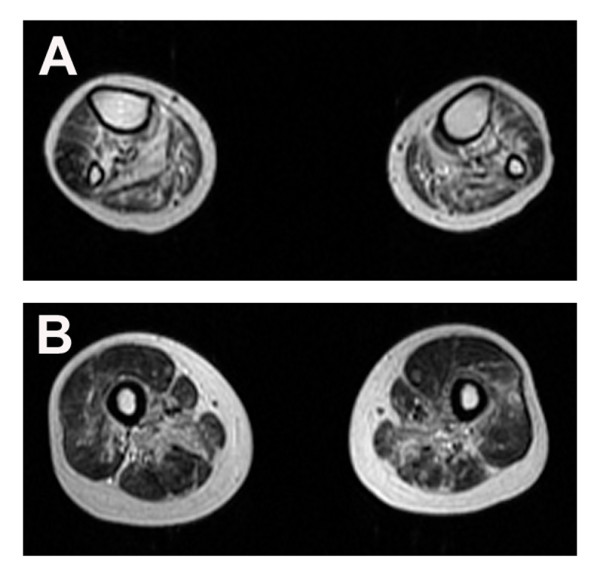
**Muscle magnetic resonance imaging.** T2-weighted magnetic resonance images of the legs (A) and thigh (B) show severe muscle atrophy and increased signal intensities in the tibialis anterior and posterior, adductor longus and magnus, and biceps femoris muscles.

A 20-year-old man was admitted to our hospital with gait disturbance. He had no family history of neuromuscular disorders. He first noticed leg dragging at the age of 14. Neurological examination showed marked weakness and atrophy in the distal leg muscles and moderate atrophy in the proximal leg muscles. Cranial muscles were not involved and sensory examination was unremarkable. No deformity of the spine and feet was noted. Serum creatine kinase level was 599 IU/L (normal, 62–287 IU/L) with no other routine biochemical abnormalities including renal, liver, and thyroid functions and a battery of immunological markers (antinuclear antibody, rheumatoid factor, anti-DNA antibody, Jo-1, etc) were all negative. Electromyography showed myogenic changes with some neurogenic changes in all muscles tested. Nerve conduction studies were unremarkable. Muscle magnetic resonance imaging studies showed atrophies and degenerations in the muscles of both anterior and posterior compartments of the legs (Figure 1A). The hamstrings were markedly involved muscles while the vastus lateralis, vastus intermedius, and vastus medius were moderately involved muscles (Figure 1B). Mutational analysis of *GNE* revealed a homozygous 1714 G>C mutation.

Muscle biopsy samples were taken from the left quadriceps femoris, and the tissue specimens were immediately frozen in isopentane chilled with liquid nitrogen. Serial 10-μm-thick transverse sections cut using a cryostat were stained by routine muscle histochemical methods. In addition, we performed immunohistochemical and immunofluorescence studies (Additional file 1 shows these methods in more detail).

**Figure 2:**
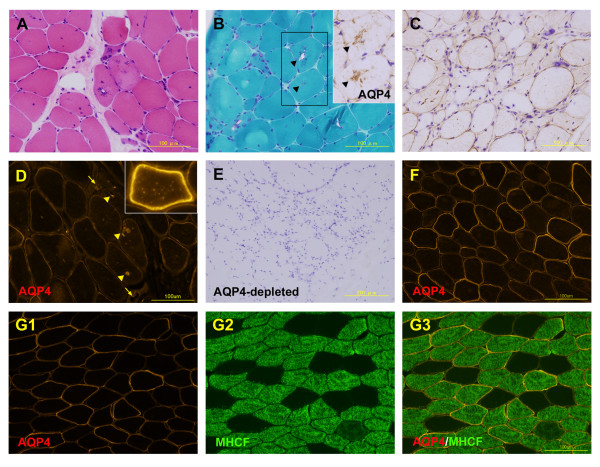
**Pathology of muscle.** Hematoxylin and eosin staining (A). Modified Gomori-trichrome staining (B) and immunohistochemical examination of aquaporin-4 (AQP4) (inset) in the serial section (Note the arrow-heads). Immunohistochemical examination of AQP4 (C). Immunofluorescence analysis of AQP4 shows that the rimmed vacuoles (RVs) and the areas surrounding the RVs are often strongly AQP4 positive (D, arrow-heads) but not always so (D, arrow). Negative controls, without incubations with anti-AQP4 antibody (E). AQP4 expression in normal control muscle (F). Double immunofluorescence analysis of AQP4 and myosin heavy chain-fast (MHCF) in the normal control muscle (G1-G3).

Cryostat sections stained with hematoxylin and eosin showed an increased variation in muscle fiber sizes, angular atrophic fibers, central nuclei, and RVs with no inflammatory cell infiltrates (Figure 2A). The RVs and areas surrounding the RVs showed intense AQP4 immunoreactivity, but the centers of the vacuoles were devoid of immunoreactivity (Figure 2B, inset), while many muscle fibers showed weak sarcolemmal AQP4 immunoreactivity (Figure 2C). Immunofluorescence analysis of AQP4 also showed that the RVs and the areas surrounding the RVs were often strongly AQP4 positive (Figure 2D). Further, strong cytoplasmic or cytosolic punctate AQP4 expression was occasionally observed (Figure 2D, inset). Negative controls, which were not incubated with anti-AQP4 antibody, showed no immunoreactivity (Figure 2E). In a control muscle sample without evidence of neuromuscular disorders, AQP4 immunostaining clearly showed the mosaicism in the AQP4 expression on the myofiber surface (Figure 2F). RVs with intensive AQP4 expression and sarcoplasmic AQP4 aggregates were never seen in the control muscle. Moreover, double immunofluorescence analysis of AQP4 and myosin heavy chain-fast (MHCF) showed that AQP4was predominantly expressed in the fast-twitch type 2 fibers (Figure 2G1-G3).

**Figure 3:**
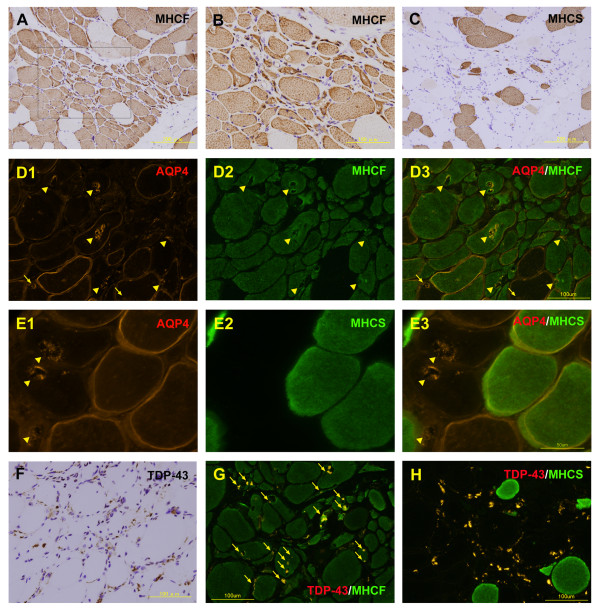
**Fast-twitch type 2 fibers are predominantly involved with strong aquaporin-4 (AQP4)-positive rimmed vacuoles (RVs) and TAR-DNA-binding protein-43 (TDP-43) aggregation. **Immunohistochemical examination of myosin heavy chain-fast (MHCF) and myosin heavy chain-slow (MHCS) in serial sections (A-C, B is high-power view of A). Double immunofluorescence analysis of AQP4 and MHCF showed many RVs with intense AQP4 expression in the MHCF-positive fibers (D1-D3, arrow-heads). Occasionally, the RVs with AQP4 expression were detected in the MHCF-negative fibers (D1 and D3, arrows). Double immunostaining of AQP4 and MHCS showed that RVs with strong AQP4 expression were mostly located in the non-MHCS fibers (E1-E3, arrow-heads). Immunohistochemical analysis of TDP-43 (F). Double immunostaining of TDP-43 and MHCF revealed that the TDP-43 immunoreactivity is mostly present in MHCF fibers (G, arrows), not in the MHCS fibers (Fig. H).

We next determined whether AQP4 expression was associated with the type of fiber in the DMRV/hIBM patient. RV-positive muscle fibers were predominantly present in MHCF fibers, whereas only some were found in myosin heavy chain-slow (MHCS) fibers in serial sections (Figure 3A- 3C). Double immunofluorescence analysis of AQP4 and MHCF showed that RVs with strong AQP4 expression were localized predominantly in the MHCF fibers (Figure 3D1-D3). In addition, double immunostaining of AQP4 and MHCS showed that RVs with strong AQP4 expression were mostly located in the non-MHCS fibers (Figure 3E1-E3). Furthermore, numerous TDP-43 immunostained aggregates were detected in many muscle fibers (Figure 3F). Double immunostaining of TDP-43 and MHCF or MHCS revealed that the TDP-43 immunostaining was most commonly present in the MHCF-positive fibers (Figure 3G), not in the MHCS fibers (Figure 3H).

**Figure 4:**
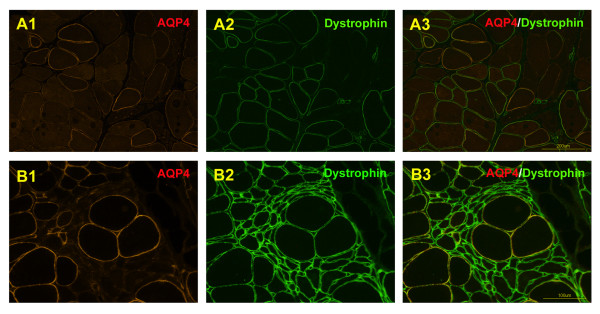
**Double immunofluorescence analysis of aquaporin-4 (AQP4) and dystrophin in a female carrier of Duchenne muscular dystrophy (DMD) and an amyotrophic lateral sclerosis patient (ALS).** A female carrier of DMD showed a mosaic pattern of dystrophin-positive and dystrophin-negative fibers on sarcoplasmic membrane with a markedly increased variability of muscle fiber diameters, and the stainability of AQP4 was strikingly patchy; some large fibers showed positive staining for AQP4 whereas almost all the small fibers were negative (A1-A3). The muscle specimens of an ALS patient showed that AQP4 immunoreactivity was weak or negative on the myofiber surface with group atrophy, but dystrophin was clearly visualized on the denervated muscles (B1-B3).

Subsequently, we performed a disease-control study of muscle AQP4/dystrophin expression. RVs with intensive AQP4 expression and sarcoplasmic AQP4 aggregates frequently seen in the DMRV/hIBM patient were not found in a female carrier of Duchenne muscular dystrophy (DMD) or in an ALS patient (Figure 4). As expected, both of the disease-controls showed some lack of AQP4/dystrophin expression in the sarcolemma, as described elsewhere [8-10].

## Discussion

To the best of our knowledge, this is the first report on AQP4 expression in the skeletal muscle of a DMRV/hIBM patient. We showed that sarcolemmal AQP4 immunoreactivity was reduced in many muscle fibers of the DMRV/hIBM patient, a female carrier of DMD, and an ALS patient. It is interesting to note that AQP4 staining was markedly increased in the areas of the RVs and in some muscle fibers in DMRV/hIBM. The most striking findings were that the fast-twitch type 2 fibers were predominantly involved with the strong AQP4-positive RVs and TDP-43 aggregations in DMRV/hIBM.

Our DMRV/hIBM case showed unusual involvement of the quadriceps muscles. In general, even at the advanced stage of DMRV/hIBM, quadriceps muscles are relatively spared. However, an earlier study has described a DMRV/hIBM patient with weakness of quadriceps [13]. Intriguingly, the study also reported that a non-DMRV/hIBM patient with predominant proximal muscle weakness of the lower extremities had *GNE* mutations [13]. On the other hand, quadriceps muscles of *GNE* knockout mouse are preferentially involved [14]. It remains unknown why clinical variations are observed in *GNE*-mutated DMRV/hIBM. Further studies are needed to clarify the genotype/phenotype correlations in DMRV/hIBM patients.

Our results about downregulation of AQP4 are consistent with the results reported in other studies [8-10]. We suppose that AQP4 downregulation occurs as a common pathway in muscle degeneration at a late stage. An important finding of our study is the result of AQP4-positive staining. We show that AQP4 staining was markedly increased at the RVs or the areas surrounding the RVs and in some muscle fibers in our DMRV/hIBM patient. Furthermore, fast-twitch type 2 fibers were predominantly involved with the strong AQP4-positive RVs, while these findings were not observed in a control muscle samples without evidence of neuromuscular disorders and in disease-control muscles. The lysosomal system is thought to be activated in DMRV/hIBM muscle because of accumulation of various lysosome-related proteins in the RVs [14,15]. Moreover, several sarcolemmal proteins, α-dystroglycan, β-dystroglycan, and α-sarcoglycan are also accumulated in the myofibers of the DMRV/hIBM mouse model, presumably because of abnormal protein misfolding/aggregation [14,15]. We surmise that the AQP4-positive aggregates in the sarcoplasm and intense AQP4 expression with RVs are associated with the lysosomal autophagic process. On the other hand, the slow- to fast-twitch conversion of soleus fibers under muscle unloading is associated with AQP4 expression in rats [16]. The fact that the modulation of AQP4 expression is associated with the transition of muscle fiber type indicates that AQP4 is an important muscle protein involved in muscle plasticity. In addition, AQP4 may protect against muscle damage, by maintaining muscle volume regulation and muscle osmolarity [6]. Thus, we consider that muscle adaptation from type 1 to type 2 fibers is associated with the change in AQP4 expression against muscle degeneration in DMRV/hIBM. A critical feature in terms of involvement of a specific type of muscle fiber in DMRV/hIBM is that the fast-twitch fibers are predominantly affected [17]. As shown in our DMRV/hIBM patient, the fast-twitch type 2 fibers would be more involved with TDP-43 accumulation in the late stage. TDP-43 positive aggregates have been observed in various vacuolar myopathies, which suggests that TDP-43 accumulation is more likely to be a common endpoint of vacuolar muscle degeneration [11].

## Conclusions

In conclusion, we found that many muscle fibers of a patient with DMRV/hIBM showed a reduction of sarcolemmal AQP4 immunoreactivity, while fast-twitch type 2 fibers were predominantly involved with strong AQP4-positive RVs and TDP-43 aggregation. Although the functional role of AQP4 in skeletal muscle is not well understood, AQP4 might be crucial in determining the survival or degeneration of fast-twitch type 2 fibers in DMRV/hIBM.

## Consent

Written informed consent was obtained from the patient for publication of this Case report and any accompanying images.

## Competing interests

The authors declare that they have no competing interests.

## Authors’ contributions

AH conceived the study and drafted the manuscript. In addition, he reviewed medical report of the patients and analyzed the neuropathological findings. KS and ST contributed to the analysis of muscle pathology. TY and YU are senior authors and oversaw all aspects of the paper, including a careful review of the final product. KS performed routine muscle histochemistry, immunohistochemistry, and immunofluorescence of the patients. All authors read and approved the final manuscript.

## Pre-publication history

The pre-publication history for this paper can be accessed here:

http://www.biomedcentral.com/1471-2377/12/22/prepub

## Supplementary Material

Additional file 1 Method of immunohistochemical and immunofluorescence study. Click here for file
