# Supplementary material for: Aquaporin-4 expression in distal myopathy with rimmed vacuoles
Source: BMC Neurol. 2012 Apr 27;12:22. doi: 10.1186/1471-2377-12-22 (PMC3477015; doi:10.1186/1471-2377-12-22)
Supplement: Additional file 1 — Method of immunohistochemical and immunofluorescence study. [file 1471-2377-12-22-S1.doc]

Method of immunohistochemical and immunofluorescence study:

Primary antibodies and dilutions used were rabbit anti-AQP4 polyclonal antibody (1:100; Santa Cruz), rabbit anti-TDP-43 polyclonal antibody (1:2000; Protein Tech Group), mouse anti-myosin heavy chain-slow monoclonal antibody (1:20; Leica), mouse anti-myosin heavy chain-fast antibody (1:10; Leica), and mouse anti-dystrophin monoclonal antibody (1:200; Novocastra). For immunohistochemical study, unstained frozen sections were fixed in acetone for 5 min at 4-℃. After phosphate-buffered saline (PBS) washing, the sections were blocked for 30 min at room temperature with 2.4% normal goat serum in PBS to avoid non-specific binding and then washed. Specimens were incubated overnight at 4-℃ with primary antibodies. After washing, sections were immersed for 45 min in horseradish peroxidase complex labeled polymer (Dako Cytomation, USA) which was conjugated with secondary antibodies. After PBS washes in a solution containing 3,-3’-diaminobenzidine -tetrachloride (Dotite, Kumamoto, Japan), the sections were pre-incubated for 10 min, and 30% H_2_O_2_ was then added for 5 min. For single and double immunofluorescence, the slices were fixed in acetone for 5 min at 4-℃ and were incubated in 2.4% normal goat serum for 30 min at room temperature. They were then incubated with the primary antibodies in 0.2% Triton X-100 overnight at 4-℃ and thereafter with a mixture of the secondary antibodies for 4 hours at 4-℃. The secondary antibodies and dilutions used were FITC-coupled goat anti-mouse IgG (1:100; KPL), and Cy3-conjugated donkey anti-rabbit IgG (1:100; Jackson). The reaction was stopped by several changes of PBS. The sections were then mounted on slides, and enclosed with cover glasses.
